# Linking Caregivers' Evaluation of Children's Mood to Brain Network

**DOI:** 10.1002/pchj.824

**Published:** 2025-02-01

**Authors:** Neng Chio Wong, Zhishan Hu, Wenhong Cheng

**Affiliations:** ^1^ Department of Child and Adolescent Psychiatry Shanghai Mental Health Center, Shanghai Jiao Tong University School of Medicine Shanghai China; ^2^ Neuroimaging Core Shanghai Mental Health Center, Shanghai Jiao Tong University School of Medicine Shanghai China; ^3^ Department of Psychological Medicine Shanghai General Hospital, Shanghai Jiao Tong University School of Medicine Shanghai China; ^4^ Shanghai Key Laboratory of Emotions and Affective Disorders Shanghai China

## Abstract

Using functional near‐infrared spectroscopy technique, this study identified lower brain network efficiency in children with anxiety and/or depression compared to healthy controls, with caregivers' evaluation of mood correlating with brain network efficiency.

The COVID‐19 pandemic has significantly impacted mental disorders such as anxiety and depression among children and adolescents (Figas, Giannouchos, and Crouch 2023). Caregivers, central to a child's development, play a crucial role in shaping both physical and emotional growth. Whether caregivers can accurately detect emotional symptoms in children and adolescents remains to be explored. This study aims to assess the accuracy of caregivers' evaluations of children's mood, delineate brain network efficiency alterations in patients with mental health conditions, and explore the association between the evaluation and brain network.

We recruited 66 adolescents from the Shanghai Mental Health Center and local schools, aged 9–18, diagnosed with anxiety and/or depression according to DSM‐5 criteria and the Kiddie Schedule for Affective Disorders and Schizophrenia for School‐Age Children, and without serious physical health conditions. The healthy control group had no mental disorder diagnoses. Sixteen anxiety patients (average age: 14.58 ± 1.53; 12 females), 12 depression patients (average age: 14.62 ± 1.35; all females), 27 with comorbid conditions (average age: 14.13 ± 1.70; 18 females), and 11 healthy controls (average age: 13.74 ± 1.68; 6 females) were included for analysis, with no significant age differences between groups. Due to sample limitations, we conducted a statistical power analysis. For comparisons between patient and control groups, the average statistical power based on Cohen's d was 0.595, indicating reasonable power in most cases to support the study's conclusions.

The study began with an 11‐min resting state scan, followed by a 7‐min, 50‐s emotional response video, and a cognitive test (Vanderwal, Eilbott, and Castellanos 2019). We assessed participants' anxiety and depression severity through the Screen for Child Anxiety Related Emotional Disorders (SCARED; Birmaher et al. 1997), the Depression Self‐Rating Scale for Children (DSRSC; Birleson et al. 1987), and the caregivers' single‐item measure (Turon et al. 2019). Participants and their legal guardians gave informed consent before the experiment. The Shanghai Mental Health Center Ethics Committee approved the study (Reference Number 2020‐09).

We measured cortical changes in oxyhemoglobin (HbO) and deoxyhemoglobin (HbR) concentrations using the NirSmart‐6000A functional near‐infrared spectroscopy (fNIRS) system at an 11 Hz sampling rate during resting and movie‐watching conditions (Plichta et al. 2006). The system's 21 emitters and 13 detectors formed 41 channels (Figure 1A). The PATRIOT 3D digitizer recorded optode positions, with the NIRS_SPM toolbox in MATLAB analyzing the 3D coordinates to localize assessed cortical areas.

**FIGURE 1:**
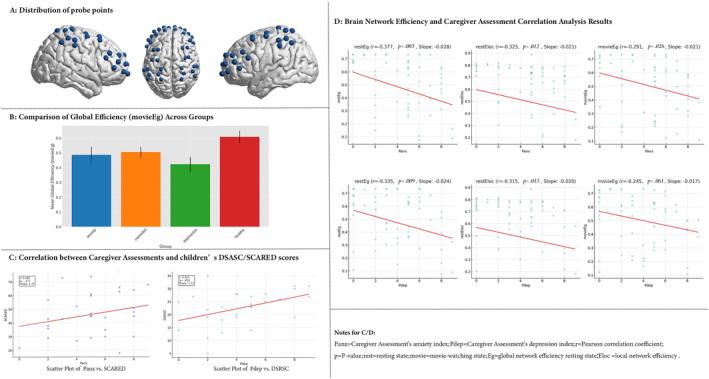
Distribution of probe points, comprehensive analysis of brain network efficiency, and caregiver assessment correlations.

Data preprocessing in MATLAB with the Homer2 package involved converting raw data to optical density, reducing global noise via principal component analysis, and correcting motion artifacts with spline interpolation. We applied a 0.01–0.08 Hz bandpass filter to resting‐state data and a 0.01‐Hz high‐pass filter to movie‐viewing data to accommodate rapid shifts. Optical density changes were then converted to HbO and HbR concentrations using the Beer–Lambert law (Scholkmann et al. 2014).

Functional brain networks were constructed through pairwise Pearson correlation analyses of the signals from the measurement channels. The GRETNA toolbox assessed topological features, including global and local efficiency, in the frontoparietal network. These metrics gauge the network's capacity for information integration across the entire network and within its local clusters, respectively (Latora and Marchiori 2001).

We examined if patients exhibited atypical brain network configurations compared to a healthy control group. Independent samples t‐tests indicated that patients demonstrated significantly reduced brain network efficiency. Notably, during the resting state, reductions were observed in both global (*t*(22.077) = −3.005, *p* = 0.007) and local efficiencies (*t*(44.484) = −3.129, *p* = 0.003). During the movie‐watching state, a decline was solely noted in global efficiency (*t*(18.841) = −2.578, *p* = 0.019).

Subsequently, ANOVA evaluated the functional differences in whole‐brain networks among the anxiety group, depression group, comorbidity group, and healthy control group (Figure 1B). No mean effect was identified.

Pearson's correlation analysis explored the relationship between caregivers' subjective assessments and standardized scale measurements, thereby assessing the accuracy of caregivers' perceptions of children's emotional and psychological states. Findings, as depicted in Figure 1C, revealed a congruence between caregivers' subjective evaluations and the scale‐based assessments (DSRSC) of children's depression, indicating partial reliability of caregiver insights. No significant correlation was found with the SCARED scale. This suggests that caregivers may be more attuned to symptoms of depression, which are often more overt than anxiety.

After that, we examined if caregivers' assessments were associated with children's brain networks. Caregivers' assessments of anxiety and depression significantly correlated with global and local brain network efficiency in healthy controls during rest and movie‐watching. However, for patients, only the comorbid group showed a negative correlation between global/local network efficiency and caregivers' assessment of depression.

We pooled the data together to examine the relationship between brain network and caregivers' assessments (Figure 1D). We observed that during rest, brain network efficiency was inversely correlated with caregiver reports. In the context of movie‐watching, a significant negative correlation was noted solely between global efficiency of the brain network and caregivers' reports of anxiety.

It can be concluded that at rest, patients' brain functions suggest deviations from the norm, even without specific tasks. A robust correlation between brain network and caregivers' assessment for healthy controls was found, while this association only existed in resting state for the comorbid group. This suggests that caregivers are particularly attuned to the emotional states of healthy children, whether at rest or movie‐watching. However, when children showed comorbid symptoms, caregivers' assessments aligned more closely with children's changes in brain function, particularly in the context of depression. Pooled data also indicated that caregivers' assessments were linked to alterations in brain network efficiency. The varying degrees of association between fNIRS metrics and caregiver assessments highlighted the complexity of brain–behavior relationships, warranting a nuanced approach to interpreting neuroimaging data in the context of mental health disorders. This study has a gender bias, as all participants in the depression group were female, which may limit the generalizability of the findings. Future research should consider gender as a factor to better explore its impact on brain network efficiency and caregiver evaluations.

## Conflicts of Interest

The authors declare no conflicts of interest.
